# PKM2 Is a Potential Diagnostic and Therapeutic Target for Retinitis Pigmentosa

**DOI:** 10.1155/2021/1602797

**Published:** 2021-11-11

**Authors:** Peiwen Zhu, Qian Yang, Gang Li, Qing Chang

**Affiliations:** Department of Ophthalmology and Vision Science, Eye and Ear, Nose and Throat Hospital of Fudan University, Shanghai 200031, China

## Abstract

Retinitis pigmentosa (RP) is a major cause of blindness that is difficult to diagnose and treat. PKM2, a subtype of pyruvate kinase, is strongly associated with oxidative stress and is expressed in photoreceptors. We investigated whether PKM2 reduces photoreceptor cell apoptosis and evaluated possible antiapoptotic mechanisms in RP. We established RP models by exposing 661W cells to blue light and modulated PKM2 activity using a PKM2 inhibitor. We measured the apoptosis rates using calcein-acetoxymethyl ester/propidium iodide double staining and Cell Counting Kit-8, the oxidative stress levels using a reactive oxygen species assay, and the changes in protein expression by western blotting. Photodamage increased PKM2 expression, cellular oxidative stress, and apoptosis of 661W cells. PKM2 inhibition significantly reduced the levels of apoptosis and oxidative stress induced by photodamage. Our data suggest that PKM2 is a potential disease marker and therapeutic target for RP.

## 1. Introduction

Retinitis pigmentosa (RP) is a set of inherited retinal diseases manifested by progressive photoreceptor cell degeneration, with an incidence of approximately 1 in 4000 [1]. The etiology and pathogenesis of RP are unknown, hindering its diagnosis [2]. Clinically, RP is mainly diagnosed by visual field imaging and electroretinography, based on highly subjective assessments, resulting in poor accuracy and reproducibility, and these factors also make it difficult to diagnosis RP at an early stage [3]. The treatment of RP has also progressed slowly due to the difficulties in diagnosis and unclear pathogenesis. Numerous studies of RP have focused on gene therapy, and many breakthroughs have been achieved in recent years. However, there is extensive genetic heterogeneity in RP, with dozens of gene mutations accompanied by X-linked inheritance and autosomal recessive genes, which make it difficult to apply gene therapy universally in the clinic [2]. Thus, finding alternative diagnostic and treatment targets for RP lesions is of particular clinical importance.

The clinical manifestations of RP include night blindness in adolescence, followed by progressive visual field constriction, and loss of visual acuity in both eyes, and ultimately blindness because the lesions in rod cells usually precede those in cone cells [4, 5]. Although the pathogenesis of RP is not well defined, apoptosis of photoreceptor cells appears to be a common feature of this group of diseases [6], and it may be accelerated by oxidative stress and light exposure [4, 5]. The retina consumes a large amount of oxygen and produces high levels of reactive oxygen species (ROS), particularly in photoreceptors, which are highly susceptible to ROS-induced apoptosis [7]. Understanding the mechanisms involved in photoreceptor cell death is crucial to identify and develop appropriate therapeutic strategies to preserve photoreceptors by inhibiting specific steps in these pathways.

Pyruvate kinase (PK), the rate-limiting enzyme in the glycolytic system, converts phosphoenolpyruvate to pyruvate. There are four isoforms of PK, namely, PKL, PKR, PKM1, and PKM2, which are differentially expressed in human tissues [8]. PKM2 is distributed in tissues such as the brain, liver, retina, and tumors and is the predominant isoform in tumors and the retina [9]. Researchers have long noted that in tumors and the retina, pyruvate is converted in large amounts to lactate despite the presence of oxygen, a process termed “aerobic glycolysis” or the “Warburg effect” that is closely related to PKM2 [9]. Many studies have confirmed that the regulation of PKM2 in tumor cells can protect against oxidative stress [10, 11]. Like tumor tissue, the retina has high rates of aerobic glycolysis with abundant PKM2 expression [12]. Therefore, regulation of PKM2 may also protect against antioxidative stress in the retina.

In this study, we investigated the effect of modulating PKM2 activity on light-induced cellular oxidative stress using a photoreceptor cell line *in vitro* as a model of RP to reveal the potential role of PKM2 in the photoreceptors of patients with RP.

## 2. Materials and Methods

### 2.1. Cell Culture

The mouse retinal cone cell line 661W was provided by Dr. Muayyad R. Al-Ubaidi (University of Oklahoma Health Sciences Center, Oklahoma City, OK, USA). The cells were incubated in Dulbecco's modified Eagle's medium (DMEM; Sigma-Aldrich, USA) containing 10% fetal bovine serum (FBS; Gibco, USA), 100 U/mL penicillin (Gibco, USA), and 100 *μ*g/mL streptomycin (Gibco, USA). The cultures were maintained at 37°C in a humidified atmosphere of 95% air and 5% CO_2_ and passaged by trypsinization (0.25% Trypsin-ethylenediaminetetraacetic acid; Gibco) every 2 or 3 days.

### 2.2. Light-Induced Cellular Oxidative Stress Damage in 661W Cells

The 661W cells were seeded at 1 × 10^4^ cells/well in 96-well plates and incubated for 24 h. The entire medium was then replaced with fresh medium containing 2% FBS. PKM2 inhibitor shikonin (Selleck, USA) or its solvent, dimethyl sulfoxide (DMSO; Beyotime, China), were added to the culture medium and incubated for 2 h. The final concentration of DMSO was not more than 0.1%. The cells were exposed to 6000 lux of blue light (wavelength~450 nm) for 3 h at 37°C to induce photodamage. The luminance was measured using a light meter (PP710, SanLiang, China). Calcein-acetoxymethyl ester (AM)/propidium iodide (PI) double staining, ROS assays, Cell Counting Kit-8 (CCK8) assays, and western blotting were performed after photodamage.

### 2.3. Calcein-AM/PI Double Staining

Calcein-AM/PI double staining was performed according to the manufacturer's instructions (Dojindo, Japan). At 24 h after light-induced damage, the cells were gently washed three times with PBS to remove any active esterases present in the culture medium. Then, the dye solution (50 *μ*L) was added to each well. The dye solution was prepared by adding PI solution (15 *μ*L) and calcein-AM solution (10 *μ*L) to PBS (5 mL), resulting in calcein-AM and PI concentrations of 2 and 4.5 *μ*M, respectively. After incubation at 37°C, the fluorescence of each well was measured using a fluorescence microplate reader.

### 2.4. Cell Viability Assay

A CCK8 assay kit (Dojindo, Japan) was used to measure cell viability before light-induced damage, immediately after light exposure, and at 24 h and 48 h after light exposure. The relative cell viability was calculated based on the optical density (OD) according to

(1) $$\begin{array}{c} \text{Cell viability }\left(\%\right)=\frac{\text{OD value of the experimental group}-\text{OD value of the blank group}}{\text{OD value of the control group}-\text{OD value of the blank group}}\times 100\%. \end{array}$$

Equation (1) shows the relative cell viability calculation, where OD is the optical density.

### 2.5. ROS Assays

The intracellular ROS levels were measured using a ROS assay kit (Dojindo, Japan). The kit contains highly sensitive 2′,7′-dichlorofluorescein diacetate (DCFH-DA), which is more susceptible to oxidation to fluorescent dichlorofluorescein (DCF) by intracellular ROS than normal DCFH-DA. Thus, the ROS level can be quantified by the fluorescence of DCF. At 24 h after light exposure, the cells were incubated with highly sensitive DCFH-DA for 20 min at 37°C and observed by fluorescence microscopy or measured at 488 nm excitation and 525 nm emission using a fluorescence microplate reader.

### 2.6. Western Blotting

The cells were washed twice with cold PBS and lysed for 30 min on ice with RIPA lysis buffer (Beyotime, China). The lysates were centrifuged at 12,000 g for 15 min, and the supernatants were collected. After measuring the protein concentration using a bicinchoninic acid assay (Beyotime, China), the supernatants were mixed with loading buffer (4 : 1; Beyotime, China) and heated at 100°C for 10 min. Samples (total protein: 10 *μ*g) were electrophoretically resolved on 12%–20% gradient Tris-glycine sodium dodecyl sulfate–polyacrylamide gels and transferred to polyvinylidene difluoride membranes using a Trans-Blot Turbo Transfer System (Bio-Rad, USA). The membranes were then blocked with 5% skim milk in Tris-buffered saline containing Tween for 1 h and incubated with the following antibodies: anti-nuclear factor erythroid 2-related factor 2 (NRF2; 1 : 1000; Proteintech, USA), anti-PKM2 (1 : 1,000; Abcam, USA), anti-PKM1 (1 : 1,000; Abcam, USA), anti-B-cell lymphoma-2 (BCL2; 1 : 1,000; Proteintech, USA), anti-BCL2-associated X (BAX; 1 : 5,000; Proteintech, USA), and *β*-actin (1 : 10,000, Abcam, USA). The antigen–primary antibody complexes were detected with horseradish peroxidase-conjugated secondary antibodies and developed using a chemiluminescent reagent (SuperSignal West Femto, Thermo Fisher Scientific, USA). The expression of *β*-actin was used as control. Densitometric analysis of the bands was performed with the ImageJ ver. 1.51 (National Institutes of Health, USA).

### 2.7. Statistics

Results are expressed as mean ± standard deviation or 95% confidence interval. Comparisons were made using significant differences between 2 groups were analyzed using the 2-tailed independent samples *t*-test. Analyses with multiple comparisons were carried out via 1-way ANOVA with a post hoc LSD correction. *P* < 0.05 was considered statistically significant. All statistical analyses were performed using SPSS version 20 (IBM Corp., USA).

## 3. Results

### 3.1. PKM2 Inhibition Did Not Inhibit the Viability of 661W Cells

**Figure 1:**
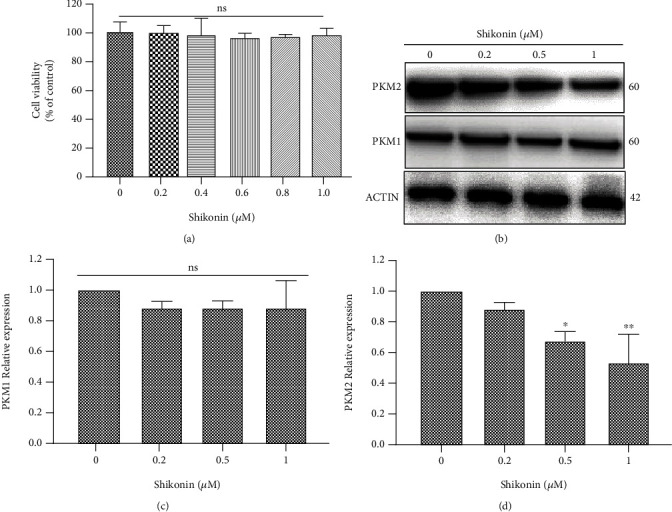
Effects of shikonin on 661W cells. (a) CCK8 assay results showing that PKM2 inhibition did not inhibit cell viability at 48 h. (b, c) Western blotting showing that shikonin did not inhibit the expression of PKM1, another PK subtype, in 661W cells. (b, d) Western blotting showing that shikonin significantly inhibited the expression of PKM2 in a dose-dependent manner in 661W cells. Notes: data from three separate experiments (*N* = 3) were averaged and analyzed by one-way ANOVA with post hoc LSD correction. ns: not significant. ^∗^*P* < 0.05 and ^∗∗^*P* < 0.01 versus the control group (0 *μ*M shikonin).

To evaluate whether shikonin may have cytotoxic effects on photoreceptor cells, we treated the 661W cells with different concentrations of shikonin for 24 h. To avoid solvent effects, the volume of DMSO administered was the same in each group and matched the volume used in the negative control group treated with DMSO alone. The results of the CCK8 assay confirmed that shikonin did not affect cell viability at any of the doses tested, and the viability of cells was equivalent to that of the negative control group (Figure 1(a)).

### 3.2. Shikonin Specifically Decreased PKM2 Expression in 661W Cells

661W cells were treated with different concentrations of shikonin for 24 h to determine its effects on the expression of PKM1 and PKM2. Western blotting showed that shikonin significantly inhibited the expression of PKM2 in a dose-dependent manner but did not inhibit the expression of another PK subtype in photoreceptors (Figures 1(b)–1(d)).

### 3.3. Blue Light-Induced Apoptosis in 661W Cells

**Figure 2:**
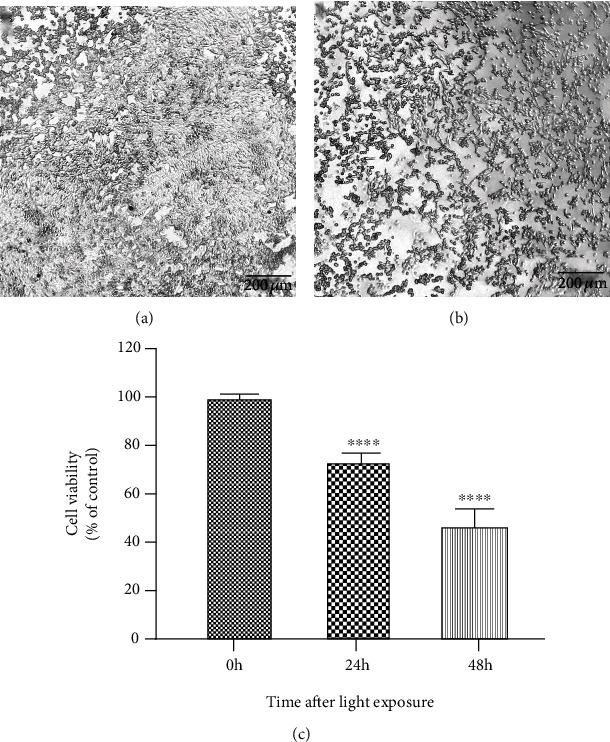
Blue light induces apoptosis in 661W cells. (a, b) Apoptosis at 24 h (a) and 48 h (b) after the 661W cells were returned to their normal culture environment following exposure to blue light. (c) Quantification of 661W cell apoptosis. Notes: data from three separate experiments (*N* = 3) were averaged and analyzed by one-way ANOVA with post hoc LSD correction. ^∗∗∗∗^*P* < 0.0001 versus the control group (0 h, before light exposure).

After exposure to blue light (6000 lux for 3 h), the 661W cells were returned to their normal culture environment for a further 24 or 48 h, at which times the extent of apoptosis was measured. The percentage of apoptotic cells was 27.82% at 24 h (95% confidence interval (CI): 25.32%–30.29%; Figures 2(a) and 2(c)) and 55.56% at 48 h (95% CI 63.43%–47.69%; Figures 2(b) and 2(c)) after light exposure.

**Figure 3:**
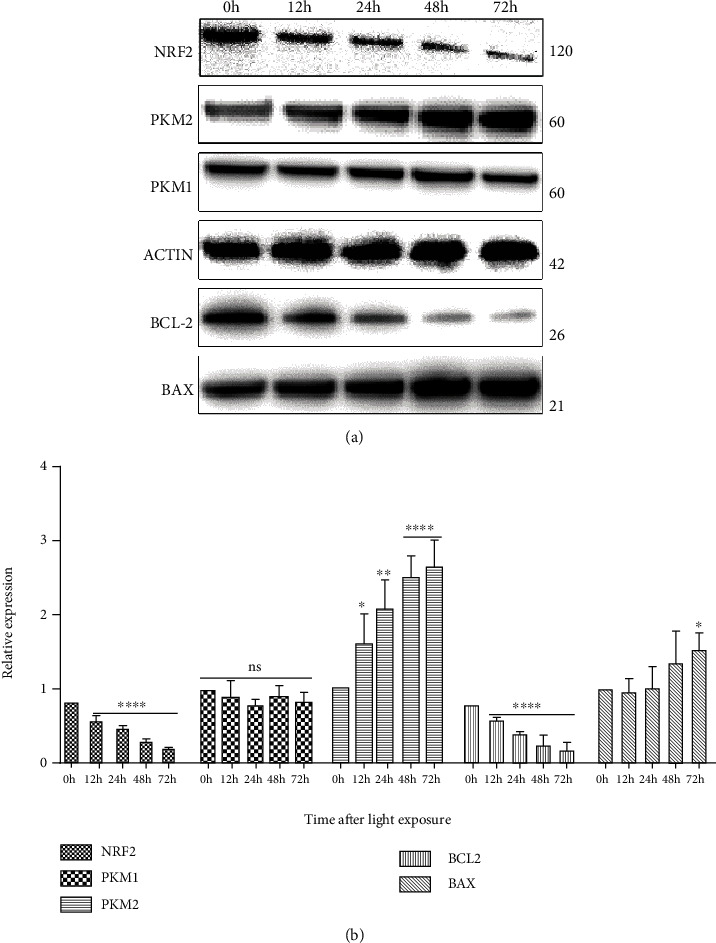
Effects of blue light exposure on protein expression in 661W cells. (a, b) Western blot analyses of NRF2, PKM1, PKM2, BCL2, and BAX expression levels in 661W cells exposed to blue light. Notes: data from three separate experiments (*N* = 3) were averaged and analyzed by one-way ANOVA with post hoc LSD correction. ^∗^*P* < 0.05, ^∗∗^*P* < 0.01, and ^∗∗∗∗^*P* < 0.0001 versus the control group (0 h).

Western blotting also confirmed that exposure to blue light-induced apoptosis in 661W cells with downregulation of BCL2 at 12, 24, 48, and 72 h and upregulation of BAX (Figure 3). At 72 h after light exposure, the relative expression levels of BCL2 and BAX were 0.16-fold (95% CI 0.04–0.27) and 1.51-fold (95% CI 1.22–1.75) the preexposure levels, respectively.

### 3.4. Blue Light Exposure Upregulated PKM2 in 661W Cells

Western blotting showed an increase in PKM2 expression over time following blue light exposure, consistent with the trend observed for BAX, whereas BCL2 and NRF2 expression levels decreased over time (Figure 3). At 72 h after blue light exposure, the relative expression levels of PKM2 and NRF2 were 2.64-fold (95% CI 2.67–3.00) and 0.19-fold (95% CI 0.16–0.22) the corresponding levels preexposure. Although there were fluctuations in PKM1 expression levels after light exposure, the changes were not statistically significant.

### 3.5. PKM2 Inhibition Reduced Apoptosis in 661W Cells following Blue Light Exposure

**Figure 4:**
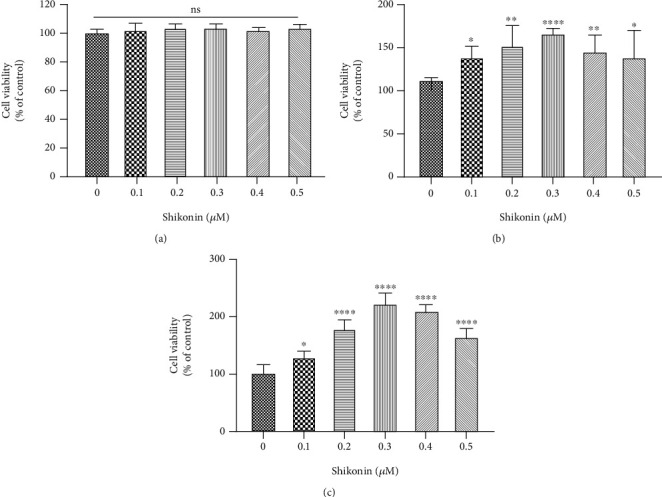
PKM2 inhibition enhances cell viability in 661W cells. (a) Effects of shikonin on the viability of 661W cells before light exposure. (b, c) Effects of pretreatment with shikonin on the viability of 661W cells at 24 h (b) and 48 h (c) after light exposure. Notes: data from three separate experiments (*N* = 3) were averaged and analyzed by one-way ANOVA with *post hoc* LSD correction. ns: not significant. ^∗^*P* < 0.05, ^∗∗^*P* < 0.01, and ^∗∗∗∗^*P* < 0.0001 versus the control group (0 *μ*M shikonin).

The 661W cells were pretreated with different concentrations of shikonin before light exposure to explore the effects of modulating PKM2 activity on apoptosis. The CCK8 assay revealed no difference in cell viability among the groups before photodamage (Figure 4(a)). Pretreatment with shikonin at concentrations of 0.1–0.5 *μ*M reduced the level of apoptosis at 24 and 48 h after light exposure (Figures 4(b) and 4(c)), with the greatest antiapoptotic effect at a concentration of 0.3 *μ*M. At this concentration, the cell viabilities at 24 and 48 h were 165.54% (95% CI 157.89–173.19) and 221.06% (95% CI 199.79–242.33), respectively, relative to the control group.

**Figure 5:**
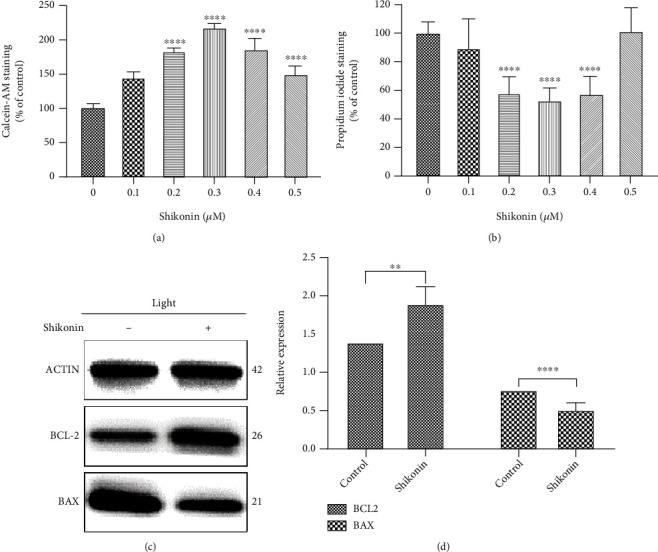
Inhibition of PKM2 reduces apoptosis induced by blue light exposure in 661W cells. (a, b) Dose-dependent effects of shikonin on apoptosis, determined by calcein-AM/PI double staining, of 661W cells at 48 h after light exposure. (c, d) Western blotting showing the effects of shikonin on BCL2 and BAX expression levels in 661W cells after light exposure. Notes: data from three separate experiments (*N* = 3) were averaged and analyzed by one-way ANOVA with post hoc LSD correction. ns: not significant. ^∗∗^*P* < 0.01 and ^∗∗∗∗^*P* < 0.0001 versus the relevant control groups (0 *μ*M shikonin).

Consistent with the results of the CCK8 assay, the calcein-AM/PI double staining revealed that the number of apoptotic cells was lowest in cells treated with 0.3 *μ*M shikonin (Figures 5(a) and 5(b)) at 48 h after light exposure. Western blotting revealed that 0.3 *μ*M shikonin upregulated BCL expression by 1.87-fold (95% CI 1.63–2.13) and downregulated BAX expression by 0.49-fold (95% CI 0.38–0.61) (Figures 5(c) and 5(d)).

### 3.6. PKM2 Inhibition Relieves Oxidative Stress in 661W Cells

**Figure 6:**
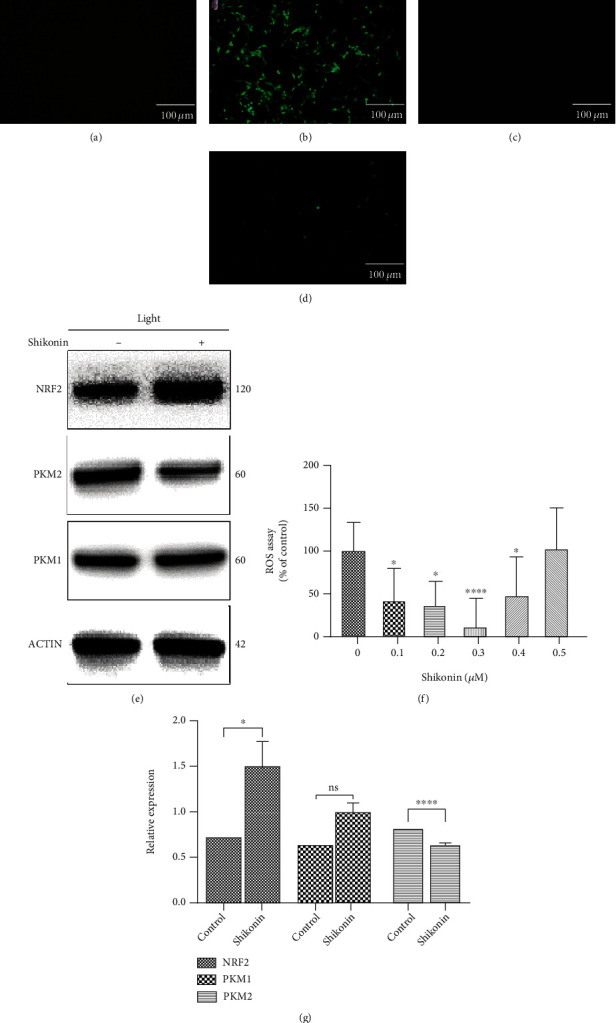
PKM2 inhibition relieves oxidative stress in 661W cells. (a–d) ROS fluorescence staining of 661W cells before blue light exposure (a), 661W cells treated with shikonin before light exposure (b), 661W cells exposed to blue light (c), and 661W cells treated with shikonin and exposed to blue light (d). (f) Quantitative analysis of ROS in 661W cells treated with different concentrations of shikonin and exposed to blue light. (e, g) Western blotting of NRF2, PKM1, and PKM2 protein expression levels in 661W cells treated with shikonin and exposed to blue light. Notes: data from three separate experiments (*N* = 3) were averaged and analyzed by one-way ANOVA with *post hoc* LSD correction. ns: not significant. ^∗^*P* < 0.05, ^∗∗∗^*P* < 0.001, and ^∗∗∗∗^*P* < 0.0001 versus the relevant control group (0 *μ*M shikonin).

Qualitative and quantitative detection of ROS showed that 0.3 *μ*M shikonin reduced the level of oxidative stress by 10.29% (95% CI 0–45.11) relative to the positive control group at 24 h after light exposure (Figures 6(a)–6(d) and 6(f)). Western blotting showed that 0.3 *μ*M shikonin downregulated the PKM2 expression by 0.63-fold (95% CI 0.61–0.66) and upregulated NRF2 expression by 1.50-fold (95% CI 1.21–1.80) at 24 h after light exposure (Figures 6(e) and 6(g)).

## 4. Discussion

RP is difficult to diagnose, especially at an early stage. RP lesions affect the retinal blood vessels, photoreceptor cells, retinal pigment epithelium, and choroid, culminating in photoreceptor apoptosis [6]. In this study, we found that PKM2 expression was upregulated during apoptosis of 661W cells. PKM2 has previously been studied as a potential diagnostic or prognostic marker for various diseases [13, 14]. Our findings imply that PKM2 may serve as a disease marker and therapeutic target for RP.

Apoptosis of rod cells precedes that of cone cells in RP [15]. The apoptosis of rod cells leads to night blindness, whereas apoptosis of cone cells leads to progressive narrowing of the central visual field and ultimately blindness. This suggests that the vision and quality of life of patients with advanced RP are highly dependent on the rate of cone cell loss. Light exposure and oxidative stress are key factors that contribute to the progression of RP. Thus, protecting cone cells from photodamage versus oxidative stress is important for preserving vision and quality of life in patients with RP [4].

661W cells are the most commonly used cell line to study RP [16]. Thus, we used this cell line to establish a model of light exposure and oxidative stress that mimics the apoptosis of cone cells in patients with advanced RP. In this cell model, apoptosis continued and the oxidative stress persisted for some time after light exposure. We found that PKM2 expression was consistently upregulated and oxidative stress was exacerbated concomitant with the apoptosis of 661W cells. Because the respiratory chain substrate end of the inner mitochondrial membrane is a major source of ROS, it is unsurprising that the flow of glucose to aerobic respiration directly increases ROS production [17]. It was previously reported that enhanced aerobic respiration promotes apoptosis of cone cells [18]. Considering these earlier findings together with our results, we suggest that the elevated expression of PKM2 promotes intracellular ROS production, which in turn induces apoptosis in cone cells. The changes in the expression of BCL2 and BAX support our theory.

PKM2 modulates intracellular oxidative stress and increases nicotinamide adenine dinucleotide phosphate (NADPH) production, in addition to reducing ROS generation by regulating glucose metabolism pathways [10, 11]. The activity of PKM2 is modified by succinylation, phosphorylation, and acetylation [9]. Inhibition of PKM2 leads to the accumulation of glycolytic intermediates, which prevent complete oxidative decomposition of glucose and instead redirect it to the pentose phosphate pathway (PPP). This contributes to the synthesis of NADPH and nonessential amino acids and promotes cell biosynthesis [19]. NADPH, generated by the PPP, is a component of multiple antioxidant stress systems and provides them with reducing equivalents [20, 21]. For example, the thioredoxin (TRX) system, which consists of NADPH, TRX reductase, and TRX, is a key antioxidant system involved in the defense against oxidative stress by regulating the protein dithiol/disulfide balance through its disulfide reductase activity [21]. The TRX system donates electrons to thiol-dependent peroxidases for the rapid removal of reactive oxygen and nitrogen species [21]. The TRX system also exerts antioxidative activity via DNA and protein repair by reducing the activities of ribonucleotide reductase and methionine sulfoxide reductase and by regulating a number of redox-sensitive transcription factors [21]. Similarly, the constitutive glutaredoxin (GRX) system, which comprises GRX, a protein with similar structure to TRX, is an important intracellular resistance system against ROS. The GRX system comprises systemic NADPH as well as glutathione (GSH), GSH reductase, and GRX and removes ROS via a similar mechanism to the TRX system [20]. In addition, reduced GSH can convert peroxides to water in a process catalyzed by GSH peroxidase, while NADPH reduces the oxidized form of GSH (GSSG) to GSH, and acts as an antioxidant [22]. The conversion of GSSG to GSH is an important mechanism in the retina countering oxidative stress and highlights the important role of NADPH in retinal cells [23].

PKM2 inhibition helps cells mitigate oxidative stress by activating PPP, while alleviating chronic intracellular oxidative stress by promoting the accumulation of the glycolytic intermediate, 3-phosphoglycerate [24]. 3-Phosphoglycerate can be diverted to the phosphoserine pathway for de novo synthesis of serine, which plays a crucial role in the antioxidant defense system as a precursor for GSH synthesis [25], and can also be used as a raw material for the synthesis of proteins, nucleic acids, and lipids involved in repairing oxidative stress damage [24].

In photoreceptor cells, all-*trans*-retinal is reduced to all-*trans*-retinol by retinol dehydrogenase to maintain visual sensitivity, a step that requires the reduction of NADPH [26]. Thus, NADPH appears to be vital for maintaining retinal visual sensitivity, maintaining retinal redox homeostasis, and providing reducing equivalents for lipid and protein synthesis [23, 26, 27]. Activation of the PPP pathway can also promote the synthetic repair of proteins, nucleic acids, and lipids that are damaged by oxidative stress [24]. In our study, treatment with a PKM2 inhibitor significantly reduced light-induced apoptosis of 661W cells and reduced the intracellular oxidative stress level, which was accompanied by upregulation of intracellular antiapoptotic proteins and downregulation of proapoptotic proteins. Therefore, PKM2 seems to be an important therapeutic target for retinal diseases, such as RP.

Overall, these findings support the development of PKM2 inhibitors as a promising therapeutic target for retinal diseases, such as RP. Indeed, Rajala et al. reported that the visual acuity of rod *PKM2*-knockout mice was not affected at 5 months [28]. Our experiments also showed that the moderate inhibition of PKM2 activity did not affect the viability of 661W cells, but the function of PKM2 was not restricted to glycolysis. More research is still needed before we can use PKM2 expression and inhibition for the diagnosis and treatment of RP.

## 5. Conclusions

We found that PKM2 is a key factor in RP and that regulation of PKM2 can reduce RP oxidative stress and protect photoreceptor cells. We believe that PKM2 is a potential diagnostic marker and therapeutic target for RP.

## Data Availability

All data are available from the corresponding author by request.
